# Cross-platform validation of a mouse blood gene signature for quantitative reconstruction of radiation dose

**DOI:** 10.1038/s41598-022-18558-1

**Published:** 2022-08-19

**Authors:** Shanaz A. Ghandhi, Igor Shuryak, Brian Ponnaiya, Xuefeng Wu, Guy Garty, Shad R. Morton, Salan P. Kaur, Sally A. Amundson

**Affiliations:** Center for Radiological Research, Columbia University Irving Medical Center, 630, W 168th Street, VC11-237, New York, NY 10032 USA

**Keywords:** Transcriptomics, Biomarkers

## Abstract

In the search for biological markers after a large-scale exposure of the human population to radiation, gene expression is a sensitive endpoint easily translatable to in-field high throughput applications. Primarily, the *ex-vivo* irradiated healthy human blood model has been used to generate available gene expression datasets. This model has limitations i.e., lack of signaling from other irradiated tissues and deterioration of blood cells cultures over time. In vivo models are needed; therefore, we present our novel approach to define a gene signature in mouse blood cells that quantitatively correlates with radiation dose (at 1 Gy/min). Starting with available microarray datasets, we selected 30 radiation-responsive genes and performed cross-validation/training–testing data splits to downselect 16 radiation-responsive genes. We then tested these genes in an independent cohort of irradiated adult C57BL/6 mice (50:50 both sexes) and measured mRNA by quantitative RT-PCR in whole blood at 24 h. Dose reconstruction using net signal (difference between geometric means of top 3 positively correlated and top 4 negatively correlated genes with dose), was highly improved over the microarrays, with a root mean square error of ± 1.1 Gy in male and female mice combined. There were no significant sex-specific differences in mRNA or cell counts after irradiation.

## Introduction

In the wake of a radiation event that results in the exposure of many in the human population to radiation, there will be a need for rapid dosimetry for medical management and triage for treatment^1–5^. Biological dosimetry has been shown to be very useful in this regard and the best-characterized methodology is the gold standard cytogenetic measurement of double strand breaks using the dicentric assay^4,6^. Other assays are being developed, tested, and validated with the potential of shortening the time-to-result. Molecular assays, such as gene expression measurements, are among the most promising approaches to bring this critical time down from days to a few hours after sampling^4^. They also reduce the large-scale equipment and space needed for high throughput cytogenetic biodosimetry^7,8^. Many laboratories have tested gene expression in human and mouse blood to develop panels of gene targets in mRNA, microRNA, and non-coding RNA towards this goal of studying radiation response^9–13^. Most human studies, however, have been performed on human blood irradiated in cell cultures^14–22^ which is a powerful method mimicking much of the in vivo human blood response in an experimental set up^16,23^. However, for investigating longer term in vivo responses, the human blood culture approach has its limitations, most importantly, lack of signaling and microenvironment effects; and degeneration of the health of blood cells over several days in culture^24^. Thus, to study longer times after irradiation, we need in vivo model systems. Large and small animal models have been proposed for this purpose and used extensively following the Animal rule^25,26^ to study responses at longer times after irradiation, and enable studies of protracted exposures, internal emitters and more complicated scenarios involving partial body exposures^27–30^.

Here, we will describe our approaches using a small animal model—the house mouse *Mus musculus* (C57BL/6N, Charles River Laboratory), for developing and validating gene expression signatures, specifically at 24 h after acute radiation exposures. Many laboratories, including ours, have published extensively on gene expression in mouse blood after irradiation (as recently reviewed^9,23^), but most of these studies focus on classifying samples as exposed versus unexposed, or above/below a threshold 2 Gy dose. We present an approach wherein we identify a set of genes that can quantitatively reconstruct doses between 0 and 8 Gy in a continuous rather than discrete manner.

We started with meta-analysis of transcriptomic microarray data in mouse blood at 24 h after irradiation, training and testing (cross validation using random separation of the data) to select about 30 genes that correlated strongly with dose and were stable over time. However, in this study we describe validation of results from the 24 h time point after irradiation as a starting point with future testing of this signature on longer time points. From this gene set we selected a smaller target set of genes to test transition to the quantitative real-time PCR platform for validation studies. Here, we used an independent cohort of animals and used a similar approach to that used to develop our human radiation-responsive gene expression signature^21^. In this approach, quantitative dose reconstruction was performed based on the difference in median signals of sets of genes that were positively or negatively correlated with radiation dose. This small-scale systems biology approach was used for dimension reduction to refine and then validate our dose reconstruction signature that consists of 7 transcripts.

## Results

### Meta-analysis of transcriptomic data and gene selection for dose reconstruction

**Figure 1:**
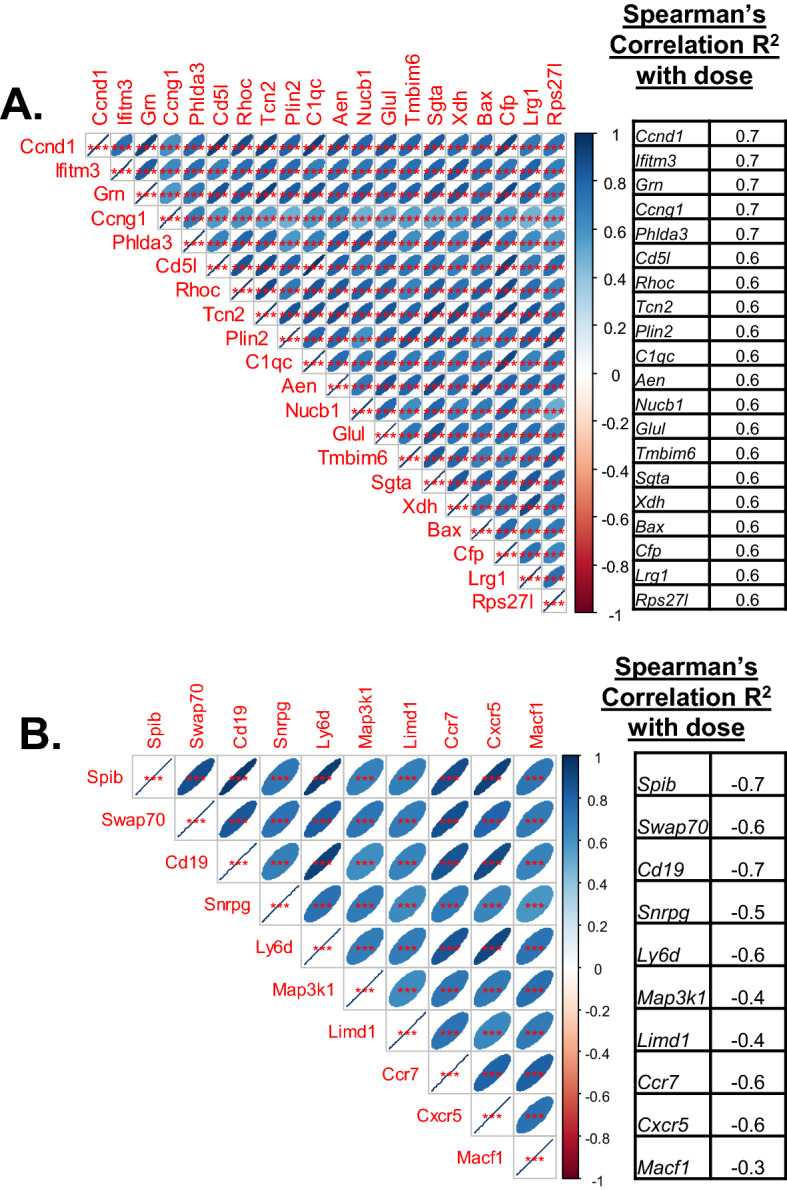
Correlation matrices of genes from the microarray meta-analysis that correlate with dose. (**A**) Genes that are positively correlated with dose, and (**B**) Genes that are negatively correlated with dose. Shown here in the matrix are pairwise correlations with each other. The size of the oval blue area indicates the correlation level, a broad oval indicates lower correlation, and the narrower oval indicates higher correlation (as indicated in the color gradient key). The table on the side shows the genes as listed and the correlation R^2^ values with dose from the microarray analyses (ranging from 0.6 to 0.7 for positive correlation and − 0.3 to − 0.7 for negative correlation). All genes in each group are highly correlated with each other indicating that the patterns are highly similar in both the up and down regulated gene groups.

**Figure 2:**
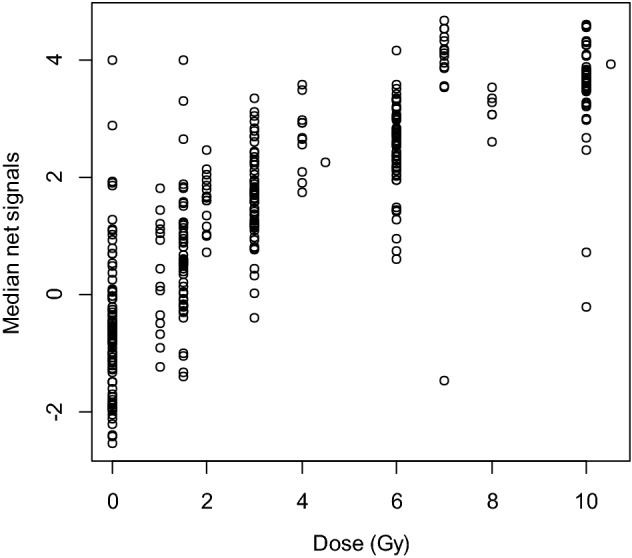
Normalized N (Net Signal) versus Dose (Gy) plots for all 30 genes included in the microarray signature. Net signal (N) is the difference between the median of the DCt of up regulated genes and down regulated genes. This value is a better indicator of dose response than either group alone, or any one gene alone, and was used for developing the model.

For this analysis, we compiled datasets from microarray analyses published by our group and others as listed in Table 1. The maximum dose was 10 Gy, and all studies used a similar acute dose rate for the irradiations, around 1 Gy/min, as described in the respective publications (see Table 1). All studies used the wild-type C57BL/6 strain. For these analyses we compiled normalized data using BRB ArrayTools^31^, and generated a table with genes as rows and samples as columns with the signal intensities of corresponding genes. We identified genes that showed a significant dose response, either positively or negatively correlated with dose. This approach resulted in a set of genes that were ranked by correlation to dose and to each other. We selected a set of 20 up regulated genes (Fig. 1A) and 10 down regulated genes (Fig. 1B) with strong dose correlations at 24 h after acute irradiation for further analyses (net_signal versus dose are plotted in Fig. 2).

**Table 1:** Microarray data used for meta-analyses with GEO accession numbers and citations.

NCBI-GEO dataset GSE#	Dose range	PMID/Citation
124612	0–10 Gy	31797975/Paul et al.^35^
196400	0, 7 Gy	NA*
62623	0–4 Gy	26114327/Paul et al.^62^
85323	0–4 Gy	28049433/Broustas et al.^63^
99176	0, 8 Gy	29351057/Rudqvist et al.^64^
114142	0, 7 Gy	31046668/Mukherjee et al.^45^
184361	0, 7 Gy	35353886/Broustas et al.^65^
52403	0–6 Gy	25255453/Lucas et al.^32^

*NA study information not available.

**Figure 3:**
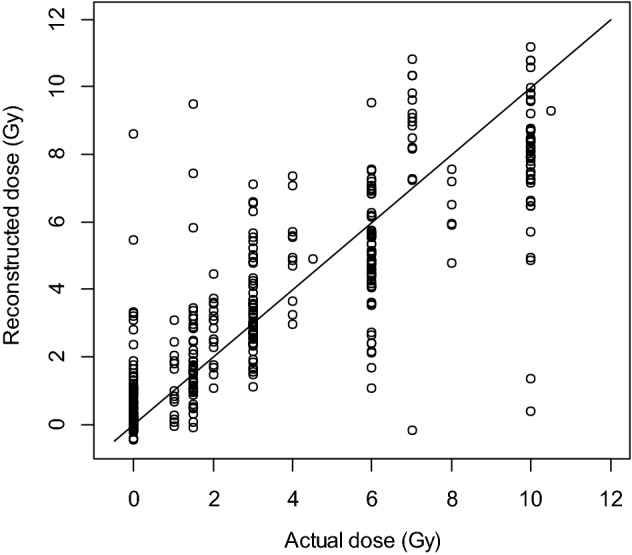
Dose reconstruction based on mouse microarray data analyzed using a nonlinear model Eq. (1). The model was based on the net gene signal (difference between median signals of the gene groups with positive and negative correlations with radiation dose), and time (days) after irradiation. The best-fit parameter values for this dose reconstruction model were: *k*_*1*_ = 0.906 (standard error SE = 0.086, *p* value =  < 2 × 10^–16^) Gy^−1^, *k*_2_ = 0.274 (SE = 0.028, *p* value =  < 2 × 10^–16^) Gy^−2^, *k*_*3*_ = 0.549 (SE = 0.069, *p* value = 1.5 × 10^–14^) days^−1/2^.

Using these genes for dose reconstruction on microarray data a robust dose response curve was generated (Fig. 2), and then used for the model for dose reconstruction (Fig. 3). Biological analysis using gene ontology and pathway analyses suggested that most of these genes are known to be involved in the DNA damage response to stress and radiation in the blood, and therefore are good candidates to measure and reconstruct dose in mice.

Next, we assessed the stability of the dose reconstruction model by 1000 random splits of the data into training/testing halves. The results of this approach gave an R^2^ value of 0.695 (standard deviation, SD 0.0388). When the data were randomly split into a training–testing set, the root mean squared error (RMSE) for the training set was 1.86 Gy (SD 0.122) and RMSE for the test set was similar at 1.9 Gy (SD 0.126). This shows that the model fits were relatively stable over random splits, but the dose reconstruction quality on these microarray data was not greatly improved (R^2^ < 0.7, RMSE ~ 1.9 Gy). Especially the day 1 samples were reconstructed with a larger error (R^2^ < 0.6, RMSE ~ 2.2 Gy). However, from these calculations we obtained a list of dose-responsive genes (up and down) that were relatively stable over time (Supplementary data table 1 lists all the genes with annotations). A subset of these genes was selected for qPCR analyses using an independently irradiated cohort of adult mice.

### Blood counts and Quantitative RT-PCR analysis in an independent mouse cohort

**Figure 4:**
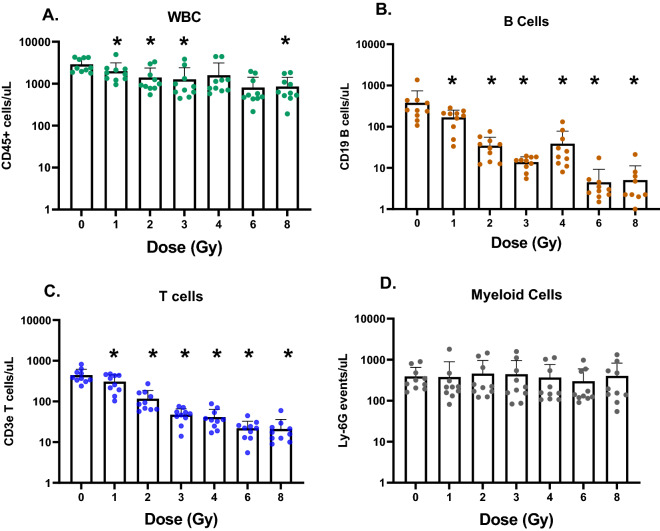
Blood cell counts from flow cytometry analysis in an independent mouse cohort. (**A**) Leucocytes (CD45 + cells)/µL of blood, (**B**) B cells (CD19 +)/µL of blood, (**C**) T cells (CD3e +)/µL of blood; and (**D**) Myeloid cells/µL of blood are plotted with dose as the independent variable. All doses of irradiation produce a significant change of T and B cell numbers compared to control levels; however, B cells are more sensitive. 5 males and 5 females were used for this analysis and data is combined, as there were no differences by sex. (*p* values are indicated in the figure as *p < 0.05).

At necropsy, an aliquot of blood was used for immunophenotyping, measuring levels of leucocytes, T and B cells and neutrophils (Fig. 4). Mouse blood cell-count data were log_n_-transformed and analyzed by linear regression, fitted using a generalized least squares method (to allow variance to depend on a power of the fitted values) using the *gls* function in *R*. The goal was to characterize the dose responses for B and T cells, and to assess potential differences in dose response parameters between males and females. Sex was included as a binary variable (Sex, males = 1, females = 0) and as an interaction term (Sex × Dose). There were no significant differences between males and females by this analysis; the Sex and Sex × Dose terms did not achieve statistical significance for either B-cells or T-cells. Supplementary Figure 1A shows the fitted parameters, along with the dose response of T cells (Supplementary Figure 1B), and B cells (Supplementary Figure 1C).

Isolated RNA was processed to cDNA and further to measure mRNA changes for genes that were selected based on signal intensity in the microarrays. For both up and down regulated groups of genes, to ensure that we were inclusive across a range of sensitivity, from low to high copy number RNA, we chose genes based on signal groupings as follows: high copy number transcripts signal intensity > 10,000; low copy number transcript signal intensity < 2000; and an intermediate group 2000 < signal < 10,000. These genes were: *Ccng1* (cyclin G1), *Aen* (apoptosis enhancing nuclease1), *Sgta* (small glutamine-rich tetratricopeptide repeat (TPR)-containing, alpha), *Grn* (granulin), *Ccnd1* (cyclin D1), *Phlda3* (pleckstrin homology-like domain, family A, member 3), *Rhoc* (ras homolog gene family, member C), *Xdh* (xanthine dehydrogenase), *Bax* (BCL2-associated X protein), *Cd5l* (CD5 antigen-like), *Tcn2* (transcobalamin 2) and *Lrg1* (leucine-rich alpha-2-glycoprotein 1) in the up regulated gene group. *Cd19* (CD19 antigen), *Cxcr5* (chemokine (C-X-C motif) receptor 5), *Ly6D* (lymphocyte antigen 6 complex, locus D) and *Ccr7* (chemokine (C–C motif) receptor 7) were in the down regulated group. Geometric mean of *Actb* (actin B) and *Gapdh* (glyceraldehyde-3-phosphate dehydrogenase) were used for all analyses.

**Figure 5:**
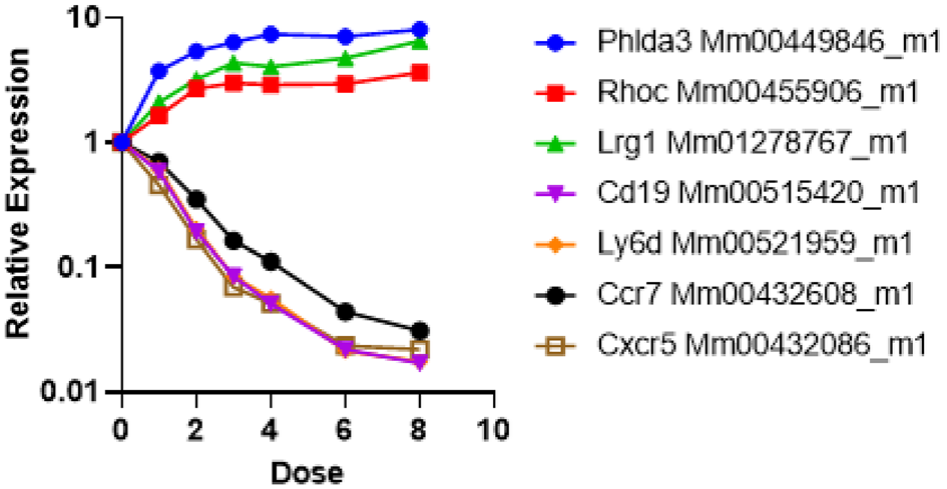
Quantitative RT-PCR results in an independent mouse cohort. Responses of male (n = 5) and female mice (n = 5) are plotted individually as a function of radiation dose for the 7-gene set measured by 2^−ΔΔCt^ method. The response of these 7 genes was the same in both sexes and is plotted separately for male and female mice in Supplementary Figure 2.

Further DCt analyses showed that *Phlda3* [correlation coefficients of − 0.6(M), − 0.7(F)], *Rhoc* [correlation coefficients of − 0.5(M), − 0.7(F)], and *Lrg1* [correlation coefficients of − 0.6(M), − 0.6(F)] were best correlated with dose among the up regulated genes tested. *Cxcr5* [correlation coefficients of 0.9(M), 0.8(F)], *Cd19* [correlation coefficients of 0.9(M), 0.9(F)], *Ly6D* [correlation coefficients of 0.9(M), 0.9(F)] and *Ccr7* [correlation coefficients of 1.0(M), 0.9(F)] gave the best dose correlations among the down regulated genes. Results of our qRT-PCR analyses for these genes with the best fold change to dose correlations combining male and female animals are shown in Fig. 5 (full table shown in Supplemental data table 3). Corresponding best-fit parameter values for the multiple regression model are shown in Table 2 (relative expression of these 7 genes compared with sham control is shown separately for males and females in Supplementary Figure 2).

**Table 2:** Assessment of sex effects on dose reconstruction using mouse qRT-PCR data. Sex was included in the multiple regression model as a binary variable: male = 1, female = 0.

Parameter	Best-fit value	Standard error	*p* value
Intercept	2.624	0.2329	< 2 × 10^−16^
N	− 0.766	0.059	< 2 × 10^−16^
N^2^	0.038	0.018	0.043
Sex × N	− 0.057	0.086	0.51
Sex × N^2^	0.033	0.031	0.28
Sex	− 0.166	0.385	0.67

**Figure 6:**
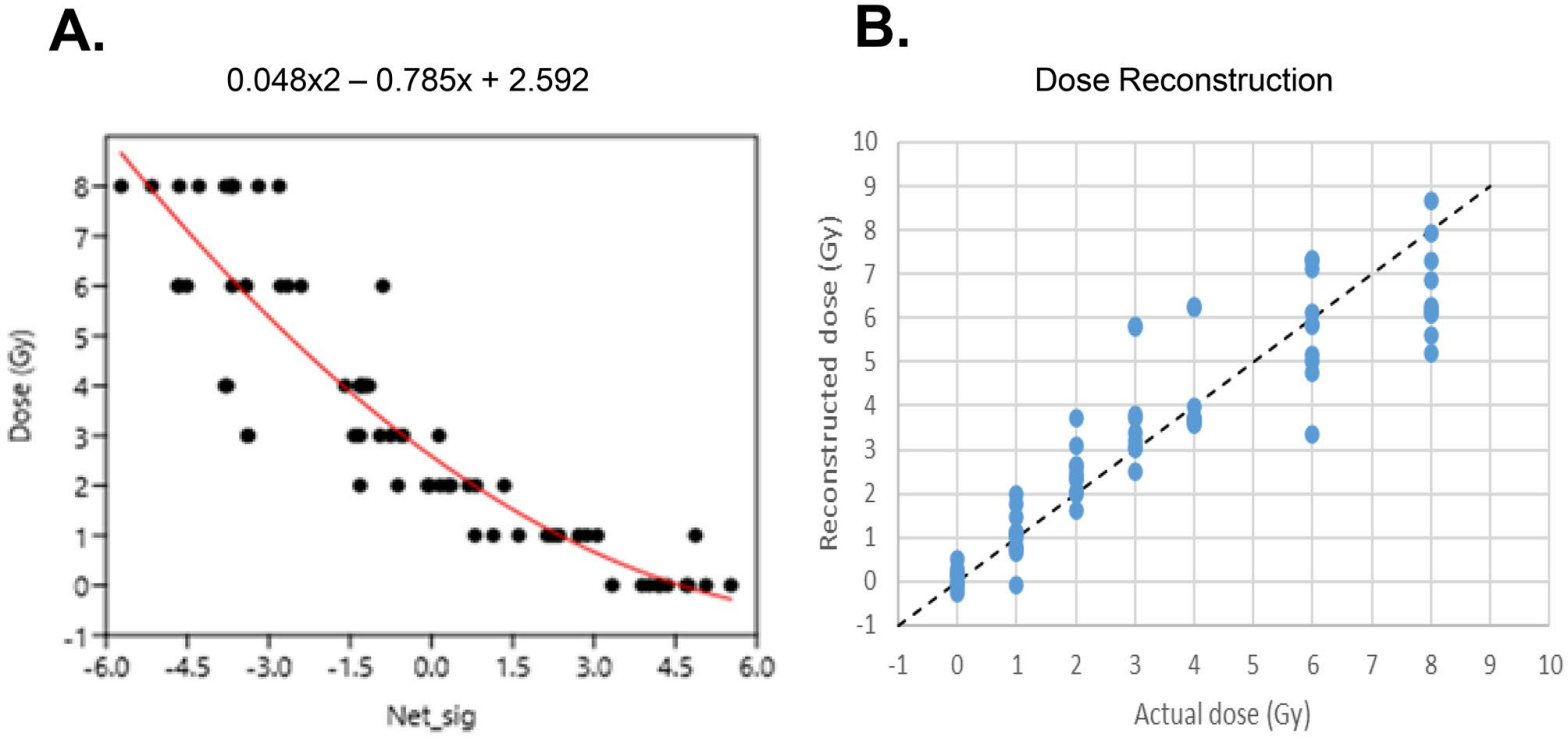
Dose reconstruction in the independent mouse cohort using qRT-PCR. (**A**) Plot of the best-fitting regression equation (**B**) plot of reconstructed dose versus actual dose, which was used to determine RMSE.

Our assessments of potential sex effects (Table 2) suggested that all terms containing Sex were not statistically significant, and they were dropped from the model (dose reconstruction for males and females is shown separately in Supplementary Figure 3). The retained simpler regression model (containing only N and N^2^) is described in Table 3. The R^2^ for dose reconstruction from this model was 0.84 and RMSE was 1.1 Gy (Fig. 6). These performance metric values suggest an improvement with qRT-PCR measurements compared to the “training” microarray data analysis where RMSE values were ~ 1.9 Gy, as described above.

**Table 3:** Best-fit parameter values for the preferred dose reconstruction model using mouse qRT-PCR data.

Parameter	Best-fit value	Standard error	*p* value
Intercept	2.592	0.181	< 2 × 10^−16^
N	− 0.785	0.042	< 2 × 10^−16^
N^2^	0.048	0.014	0.0013

## Discussion

Previous work from our group and others has emphasized the use of the mouse model as a tractable system for discovery and validation of radiation biodosimetry endpoints that cannot be studied directly using healthy humans^9,23,32–35^. With the caveat that there are species specific^36^ and even strain specific differences^37,38^ in radiation response, we trained an algorithm using the transcriptomics datasets available in the NCBI Gene Expression Omnibus database for quantitative radiation responses at 24 h. From this analysis we selected the top dose-correlated genes (positive and negative) and trained and tested our method using the net signal (N) as a correlate of dose. Typically, gene signatures that have potential for field applications are viewed to consist of a few genes to tens of genes^39–41^. Therefore, we started with 30 top ranked genes for quantitative RT-PCR testing, then further reduced the number of genes to a lower threshold, beyond which addition of more genes would not dramatically change the average error measured by RMSE.

Gene ontology using the PANTHER database for the 30 top genes from microarray data analyses suggested enrichment of biological processes related to the intrinsic apoptotic signaling pathway in response to DNA damage by p53 (Bonferroni corrected *p* value < 0.012, genes *Phlda3, Aen, Bax* and *Rps27l)* and leukocyte activation (Bonferroni corrected *p* value < 0.003, genes *Ly6D, Cxcr5, Ccr7, Cd19* and *Grn*). Further detailed analysis of the gene ontology tree indicated leucocyte activation processes specifically related to B-cell activation and differentiation (Bonferroni corrected *p* value < 0.013) were enriched. Pathway analysis using Ingenuity (IPA) also suggested that p53 signaling (*p* value < 0.0013), IL-7 signaling (*p* value < 0.0025) and interferon signaling (*p* value < 0.0009) were top signaling pathways affected by these genes. These results of biological functions represented in our signature are congruent with the major processes represented among human gene expression changes after irradiation in blood^14,16^ based on ex vivo studies. These responses, as well as p53 and DNA damage response at the mRNA level, are well established in the field of radiation biology. The interesting point here is that some of these genes are correlated with cell development and may provide an indication not only of dose but also increased damage to specific blood cell subpopulations after radiation. Additionally, the observation that immune cell changes, such as activation and differentiation are enriched biological functions among the top genes, indicates that the in vivo model captures additional information about the radiation response, which may correlate with cell depletion in the blood after increasing doses. B-cell markers *Cd19* and *Ly6D* are highly down regulated after irradiation in blood and may be indicative of cell-number changes in this cell subpopulation, shown to be radio-sensitive to acute and protracted irradiation^36,42,43^.

Next, we processed an independent cohort of male and female C57BL/6 adult mice to measure these genes using the real time qRT-PCR platform. The range of doses between 0 and 8 Gy, was similar to that in the microarray studies and whole-body irradiations were delivered at a conventional dose rate of 1 Gy/min. We measured cell counts and mRNA changes in blood at 24 h after the acute dose irradiations and compared the results and dose reconstruction across the two platforms. Both T and B cell counts in the blood dropped significantly at all irradiation doses compared with controls, however, no differences were observed by sex, even at the highest 8 Gy dose (Mann–Whitney *p* values comparing T and B cell count versus dose for males and females was ~ 0.04 at only one dose). The 7-gene signature used in this study were down selected from the results of qRT-PCR experiments and are *Phlda3*, *Rhoc* and *Lrg1* in the up regulated gene group. *Cd19*, *Cxcr5*, *Ly6D* and *Ccr7* were in the down regulated group. Among these genes *Phlda3* is a well-known radiation response gene in both normal human and cancer cells^44–46^ and a known target of p53^47^. Lrg1 protein in blood has been detected as an early survival biomarker after radiotherapy for squamous cell carcinoma^48–50^ and as a serum biomarker for radiation exposure^51^. Cd19 is a commonly used biomarker for detection of B cell development and diagnosis of leukemia^52^. *Ly6D* mRNA expression is induced by ATM and p53 after radiation in MCF10A cells^53^ and is also a surface protein involved in B cell lineage development^54^. *Cxcr5* mRNA is up regulated in T cells after antigen exposure driven by radiation-resistant cells^55^ but a role in response to radiation has not been established. *Ccr7* mRNA is down regulated in response to radiation in dendritic cells and may influence cancer cell migration^56,57^. Together, these mRNAs make a blood-based signature in response to radiation that is highly reproducible for dose reconstruction of acute radiation exposure with the low error of ~ 1 Gy with the same accuracy in both sexes. Also, we established that although -omics approaches are best at discovery, testing-training, and dimension reduction; moving to a more streamlined and targeted platform such as the well-established and widely used PCR assays gave a significant improvement of both measurement of individual genes and application of the dose reconstruction algorithm (compared in Supplementary data Figure 4). We also compared Pearson’s correlation R^2^ values measured by the microarray and qRT-PCR platforms for each gene, and for all down-regulated genes there was a significant difference (adjusted *p* value < 0.05) in qRT-PCR measurements, but no significant differences for up regulated genes.

Our approach was to start with microarray transcriptomics data, casting a wide net to capture the best radiation response genes for continuous dose reconstruction, then move to a qRT-PCR platform for independent validation, and finally narrow these down to < 10 genes that may be translatable to the field. This signature can form the basis for further experiments using the mouse model, simulating more complicated exposure scenarios with realistic parameters such as dose rate, presence of neutrons, partial body exposure and internal emitters. Such studies may provide indications for enhancement of the core 7-gene signature. Although we have separately developed a microarray-based signature for dose reconstruction in human ex vivo irradiated blood^21^, we have found that the acute photon signature has no overlap of genes between human and mouse homologs. The human homologs of the 7 mouse genes presented here do show dose responses in human blood ex vivo studies, however, they do not pass statistical significance and therefore, are not optimal for dose reconstruction.

In summary, we present here our data mining approach for identifying and independently validating a set of genes that can be used to quantitatively reconstruct dose in mouse blood cells 24 h after whole body irradiation at the dose rate of 1 Gy/min. We took advantage of the -omic data available in the literature, collated them together and statistically determined the best candidates for quantitative dose reconstruction. We tested genes on a different platform, the gold standard real time qRT-PCR, using available Taqman assays and improved the dose reconstruction by reducing error, going from an RMSE of 1.9 Gy with microarray data to 1.1 Gy with qRT-PCR. We also showed that a few genes were sufficient for dose reconstruction, 3 up- and 4 down- regulated mRNA used in combination. This core 7-mRNA signature can be further expanded to test other parameters of radiation exposure in this small animal model system.

## Methods

### Statistical meta-analysis of microarray data

Gene expression datasets were selected based on a literature search for the terms “radiation”, “biodosimetry” and “mouse blood” on the NCBI gene expression omnibus website (https://www.ncbi.nlm.nih.gov/geo/) and are listed in Table 1 with the GEO accession numbers for uploaded datasets and publication PMIDs. We started by pooling the available mouse mRNA microarray data sets using normalized and averaged signal intensities, dropping missing values wherever possible. Doses were as indicated in Table 1 (details in Supplementary data table 1). For some datasets only one non-zero irradiation dose was available, and in other cases a dose range. After merging all datasets, we retained n = 90 of 0 Gy samples; n = 14 of 1 Gy samples; n = 50 of 2 Gy samples; n = 59 of 3 Gy samples; n = 11 of 4 Gy samples; n = 60 of 6 Gy samples; n = 15 of 7 Gy samples; n = 6 of 8 Gy samples and n = 48 of 10 Gy samples. Normalization of all data was the same, using the median array as the normalizer within a data set.

Using the compiled data sets, we wrote customized code in the *R* programming language to look for genes positively or negatively correlated with radiation dose (based on Spearman’s correlation coefficients). We focused on genes that were stable over time (1–7 days, wherever the data was available) after irradiation by calculating the correlation coefficients with dose for all time points combined for each gene. We retained for further analysis only those genes whose correlation coefficients with dose were statistically significant (*p* values < 0.05 after Bonferroni correction). Training and testing split of the data was performed randomly into halves, using the caret *R* package. We focused all experiments on the 24 h time point after acute photon irradiation as the relevant early time point for biodosimetry, that is also shared with many human studies.

Since the retained genes were often strongly correlated with each other and tended to have similar dose response shapes, we did not treat each gene as a separate predictor of dose, but instead sought to combine gene signals to reduce the data dimensionality and increase the robustness of resulting dose reconstructions. We selected the top 20 genes with positive correlation with dose, and the top 10 genes negatively correlated with dose and calculated median signals in each of these two groups. The difference between group medians was treated as the net signal (N), which was then used for dose reconstruction. Sensitivity analysis showed that choosing somewhat different numbers of genes in each group (*e.g.,* 10 and 10, 30 and 30) did not substantially change the correlation of the net signal with dose.

From these calculations we obtained a list of dose-responsive genes (up and down) that were relatively stable over time (Supplementary data table 1 lists all the genes with annotations). A subset of these genes was then selected based on signal intensity range/relative copy number, for qRT-PCR analyses using an independently irradiated cohort of adult mice. For dose reconstruction, we fitted the following simple function by robust regression (using the *nlrob* function in the *R* programming language), where D is dose (Gy), N is the net gene signal (difference between median signals of the gene groups with positive and negative correlations with radiation dose, which varied from 0 to 10 Gy), T is time (days) after irradiation (which varied from 0.25 to 7 days), and *k*_1_-*k*_3_ are model parameters (see Fig. 3 legend for parameter values used):

1 $$ {\text{D}} = {\text{k}}_{1} {\text{N}} + {\text{k}}_{2} N^{2} + {\text{k}}_{3} \sqrt T $$

The structure of Eq. (1) was not mechanistically motivated, but empirically established based on examination of the data patterns. We evaluated alternative structures, where T was raised to the first or second powers instead of a power of ½, and where the T terms acted multiplicatively rather than additively. We also evaluated fitting these models by an ordinary least squares’ procedure (*nls* function in *R*) instead of by the robust procedure. This empirical exploration suggested that the structure of Eq. (1) fitted by a robust algorithm generated the best performance metrics during random training/testing splits of the data (splitting was into halves, repeated 1000 times), which are discussed in the Results section.

### Animal irradiations and sampling

All animal husbandry and experimental procedures were conducted in accordance with applicable federal and state guidelines and approved by the Animal Care and Use Committees of Columbia University (Assurance Number: A3007-01) and also in compliance with ARRIVE guidelines^58^. C57BL/6NCrl mice were purchased from Charles River Laboratories (Wilmington, MA). For validation of gene expression using the real-time qPCR method, we irradiated 5 male and 5 female young adult (~ 12 week) C57BL/6 mice to 0, 1, 2, 3, 4, 6 and 8 Gy of x rays using an XRAD-320 Biological Irradiator (Precision X-ray, North Branford, CT) at a dose rate of 1 Gy/min at the Center for Radiological Research. The irradiator is equipped with a custom-made Thoraeus filter (1.25 mm Sn, 0.25 mm Cu, 1.5 mm Al, HVL 4 mm Cu) and dose rate from the X-Rad-360 is calibrated periodically using a factory-calibrated Accudose 10 × 6–6 Ionization Chamber. 24 h after irradiation, we collected blood using cardiac puncture following euthanasia with CO_2_ and split the blood for blood counts and RNA isolation. The majority volume of blood was added to 4X volume of PAX solution (Becton Dickinson, NJ) and inverted 10X before freezing at − 80 °C. After overnight freezing, the samples were thawed at room temperature and left for > 2 h before processing for total RNA isolation using the PAXgene® Blood RNA kit (Qiagen, Valencia, CA) according to the manufacturer’s protocol. Isolated RNA was quantified using the Nanodrop One spectrophotometer (Thermofisher) and A_260/280_ ratios recorded (average yields of RNA and metrics are shown in Supplementary data table 2).

A 20 µL aliquot of whole blood was processed for immunophenotyping using a CytoFLEX flow cytometer (Beckman Coulter Inc., Brea, CA). Cell counts were quantified by standard flow cytometry methods, using antibodies specific to mouse blood cell surface antigens as follows: Neutrophils (Biolegend, catalog# 127627, Brilliant Violet 421™ anti-mouse Ly-6G), WBC (Biolegend, catalog#103115, APC/Cy7 anti-mouse CD45), B cells (Biolegend catalog#115508, PE anti-mouse CD19) and T cells (Biolegend catalog#100312 APC anti-mouse CD3ε). Flow cytometry data were analyzed with CytExpert 2.3 (Beckman Coulter Inc., Brea, CA).

### qRT-PCR analysis of dose reconstruction genes in mouse blood

We prepared complimentary DNA (cDNA) from 1 µg total mRNA using the High-Capacity® cDNA Kit (Life Technologies, Foster City, CA). Quantitative real-time RT-PCR (qRT-PCR) was performed for the selected genes using Taqman® assays (Life Technologies) using the most 3’ assays as follows: *Ccng1* (cyclin G, Mm00438084_m1 ), *Aen* (apoptosis enhancing nuclease1, Mm00471554_m1 ), *Sgta* (small glutamine-rich tetratricopeptide repeat (TPR)-containing, alpha, Mm00458535_m1 ), *Grn* (granulin, Mm00433848_m1), *Ccnd1* (cyclin D1, Mm00432359_m1), *Phlda3* (pleckstrin homology-like domain, family A, member 3, Mm00449846_m1), *Rhoc* (ras homolog gene family, member C, Mm00455906_m1), *Xdh* (xanthine dehydrogenase, Mm00442110_m1), *Bax* (BCL2-associated X protein, Mm00432051_m1), *Cd5l* (CD5 antigen-like, Mm00437567_m1), *Tcn2* (transcobalamin 2, Mm00443660_m1) and *Lrg1* (leucine-rich alpha-2-glycoprotein 1, Mm01278767_m1) in the up regulated gene group. *Cd19* (CD19 antigen, Mm00515420_m1), *Cxcr5* (chemokine (C-X-C motif receptor 5, Mm00432086_m1), *Ly6D* (lymphocyte antigen 6 complex, locus D, Mm00521959_m1) and *Ccr7* (chemokine (C–C motif) receptor 7, Mm00432608_m1) were in the down regulated group. These genes were selected based on range of signal intensity/relative transcript copy number from the arrays. Geometric mean of *Actb* (actin B) and *Gapdh* (GAPDH glyceraldehyde-3-phosphate dehydrogenase) were used for all analyses. All PCRs were performed in duplicate using 15 ng cDNA as input for standard PCR conditions. Relative fold-induction was calculated by the 2^−ΔΔCT^ method using Expression Suite software ver 1.3 (Thermofisher, http://www.thermofisher.com/us/en/home/technical-resources/software-downloads/expressionsuite-software) and Microsoft Excel software (Microsoft 365 apps enterprise)^59^.

We calculated the net signal (N; difference between average signals for genes with positive and negative correlations with dose) for the selected qPCR genes and relative quantification was calculated based on delta Ct measurements (using geomean of two stable housekeeping genes, *Actb* and *Gapdh*). In the N calculations we used geometric mean instead of median for the up and down-regulated gene groups because this marginally improved the correlation with dose. We then performed a multiple linear regression to reconstruct dose using the following predictor variables: N, N^2^, Sex (female = 0, male = 1), Sex × N, and Sex × N^2^.

### GO and pathway analyses

We used PANTHER database gene expression tools to analyze the gene lists generated by our meta-analysis of the microarray datasets^60^. Top biological processes and functions enriched among these genes were identified as those with Bonferroni corrected *p* value < 0.05. We also uploaded the lists of mouse dose response genes to Ingenuity Pathway Analysis® Software (IPA from Ingenuity®: http://www.ingenuity.com) and performed core analysis for top pathways and biological functions. We also compared gene lists using the Venny tool^61^.

### Ethics and approval and consent to participate

All animal husbandry and experimental procedures were conducted in accordance with applicable federal and state guidelines and approved by the Animal Care and Use Committees of Columbia University (Assurance Number: A3007-01).

## Supplementary Information

Supplementary Table 1.

Supplementary Table 2.

Supplementary Table 3.

Supplementary Figures.

## Data Availability

All except 1 microarray dataset used for meta-analyses in this study are publicly available in the NCBI Gene Expression Omnibus database (https://www.ncbi.nlm.nih.gov/geo/) with the processed microarray data in Supplementary data table s1. The remaining one is indicated in Table 1 with PMID/citation pending. The microarray datasets can be made available upon request.
