# Supplementary material for: Cross-platform validation of a mouse blood gene signature for quantitative reconstruction of radiation dose
Source: Sci Rep. 2022 Aug 19;12:14124. doi: 10.1038/s41598-022-18558-1 (PMC9391341; doi:10.1038/s41598-022-18558-1)
Supplement: Supplementary file 4 — Supplementary Figures. [file 41598_2022_18558_MOESM4_ESM.pdf]

Cross-platform validation of a mouse blood gene signature for quantitative reconstruction of radiation dose

Shanaz A. Ghandhi*, Igor Shuryak, Brian Ponnaiya, Xuefeng Wu, Guy Garty, Shad R. Morton, Salan P. Kaur and Sally A. Amundson

B cells

Coefficients:

	Value	Std.Error	t-value	p-value
(Intercept)	5.431439	0.2449634	22.172452	0.0000
Dose	-0.661612	0.0670307	-9.870276	0.0000
Sex	-0.454254	0.3451976	-1.315925	0.1928
Sex_Dose	0.110583	0.0917816	1.204850	0.2326

T cells

Coefficients:

	Value	Std.Error	t-value	p-value
(Intercept)	6.038787	0.14741720	40.96393	0.0000
Dose	-0.539373	0.04852854	-11.11455	0.0000
Sex	-0.400618	0.21230775	-1.88697	0.0636
Sex_Dose	0.076703	0.06827473	1.12345	0.2653

Supplementary Fig1A Comparison of B and T cell counts after radiation, effects of dose and sex only and then dose and sex together. In both cases, p-values for Sex and Sex+Dose were not significant, p values >0.05

1B

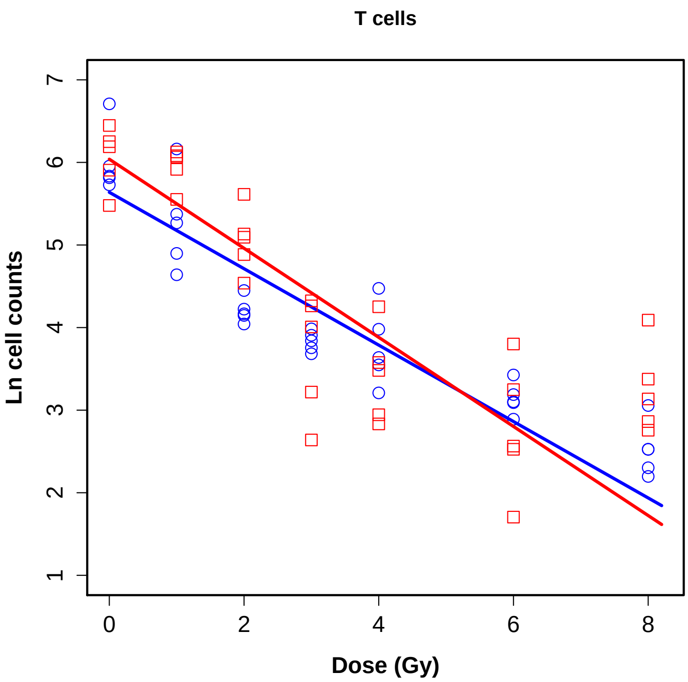

1C

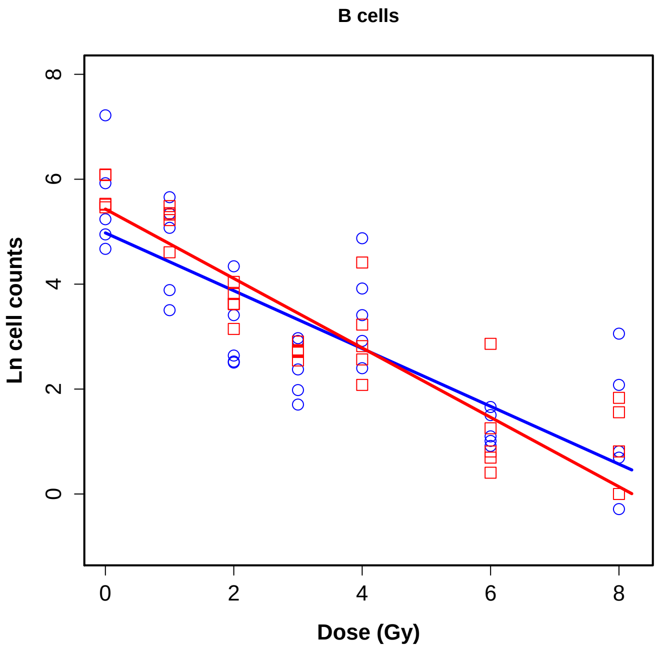

Graph shows individual data for each mouse.
Blue = males
Red = females

Supplementary figures 1B and 1C Comparison of B and T cell counts after radiation, effects of dose and sex only and then dose and sex together. In both cases, p-values for Sex and Sex+Dose were not significant, p values >0.05

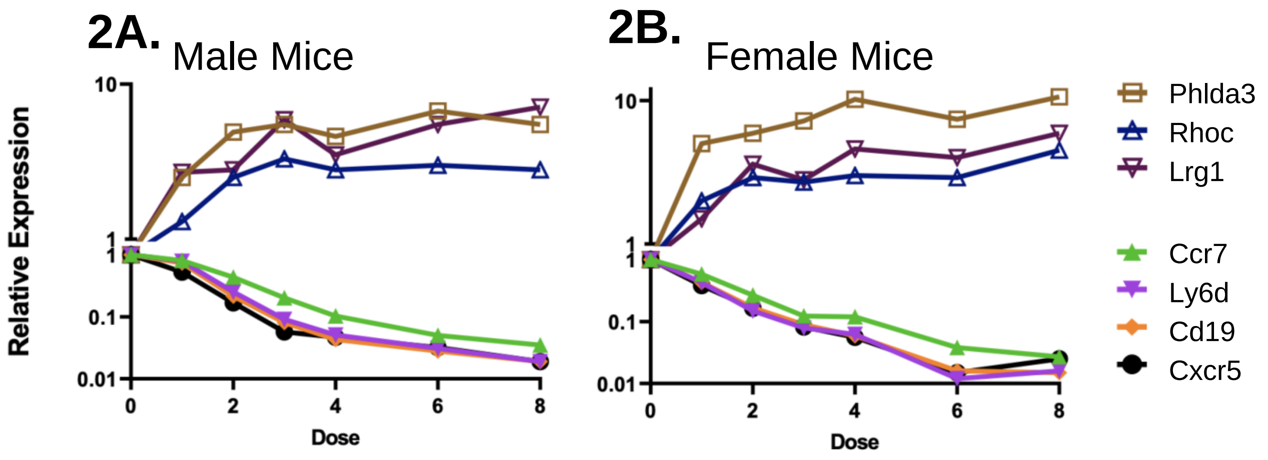

Supplementary figures 2 Relative gene expression fold changes of 7 signature mRNA by qRT-PCR , dose vs sham calibrator (using geomean of Actb and Gapdh housekeeping genes), n=5 males (**2A**) and n=5 females (**2B**).

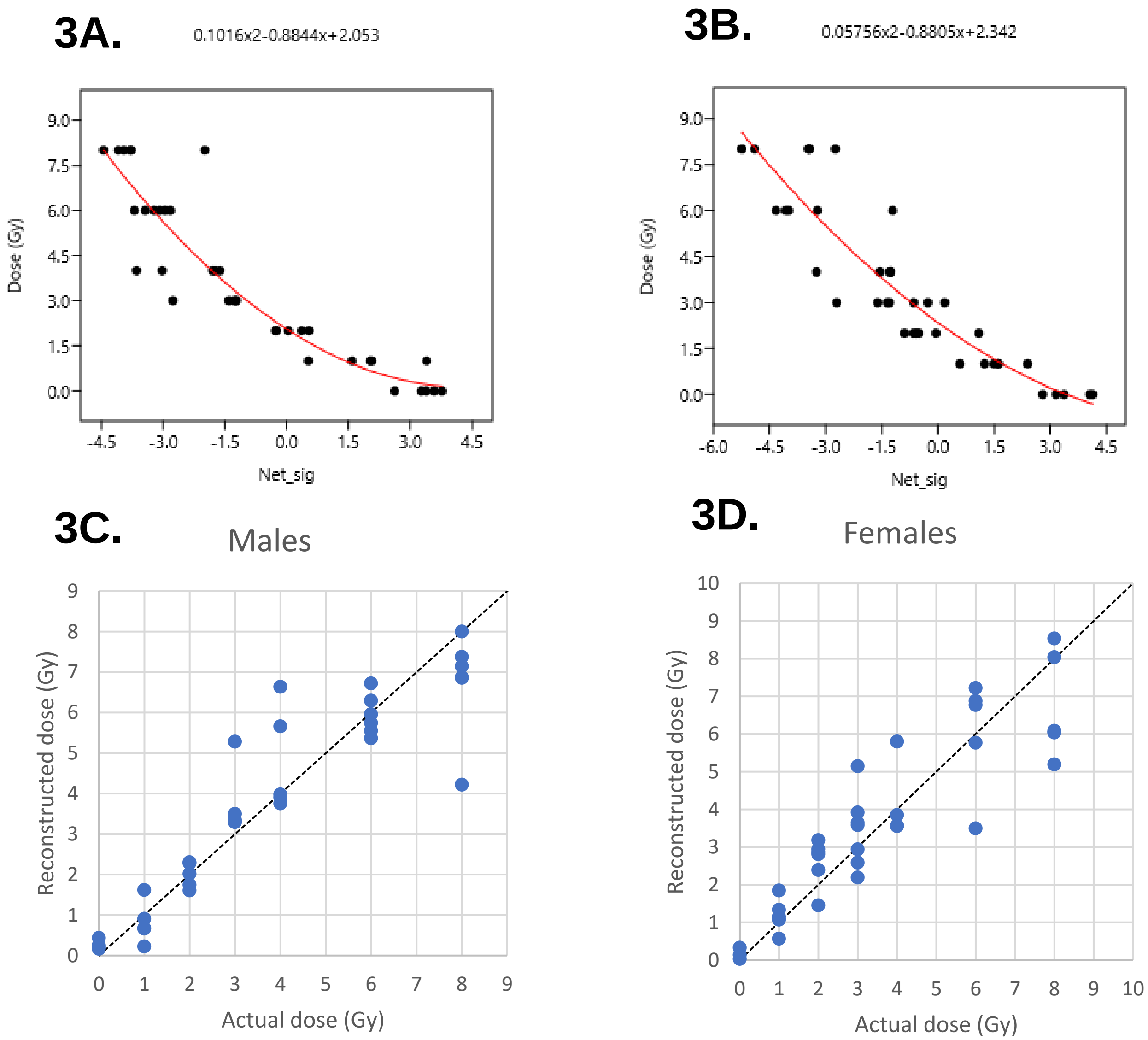

Supplementary figures 3. Male (**3A** and **3C**) gene expression results for dose reconstruction. **3A** plot Net_sig (N) independent variable and dose as dependent variable in males, and equation for the regression line. **3C** plot of reconstructed dose vs actual dose delivered and fit line using the regression model. Female (**3B** and **3D**) gene expression results for dose reconstruction. **3B** plot Net_sig (N) independent variable and dose as dependent variable in males, and equation for the regression line . **3D** plot of reconstructed dose vs actual dose delivered and fit line using the regression model.

4A. **Microarray 7genes average**

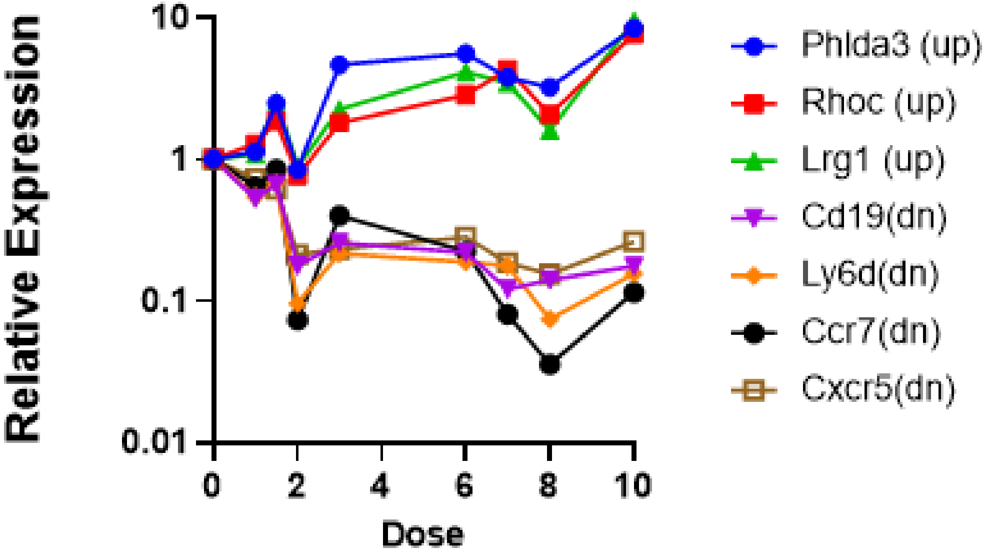

4B. **PCR 7genes average**

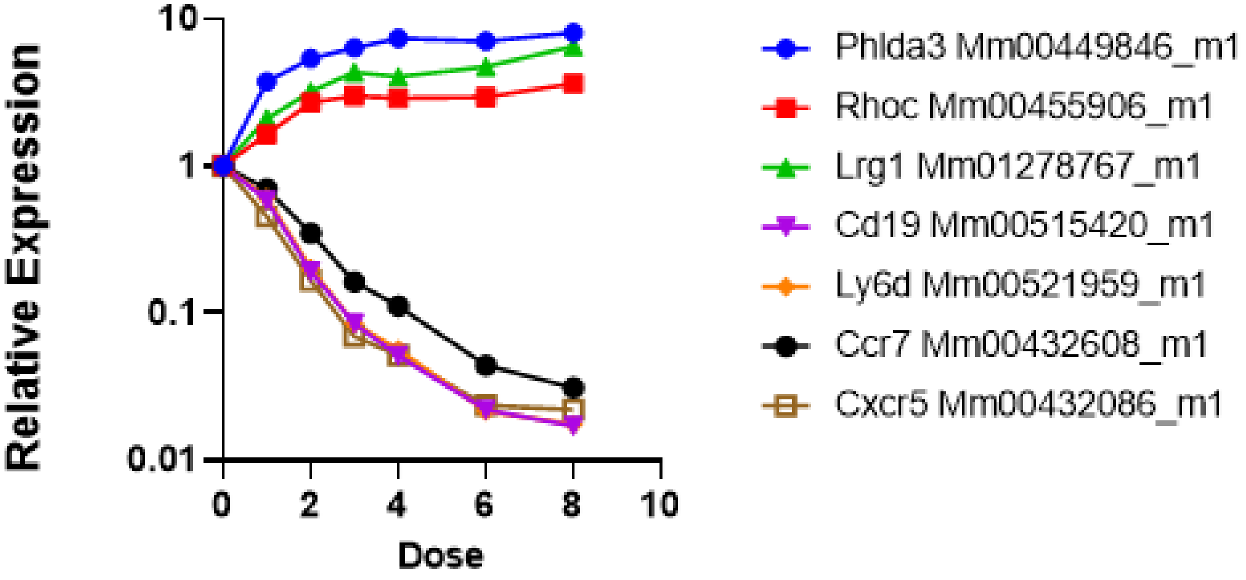

Supplementary figures 4. Relative gene expression curves, mean of n=10 (5 males and 5 females) for the 7mRNA signature genes, by microarray (**4A**) fold changes mean of irradiated vs sham, and qRT-PCR (**4B**) fold changes dose vs sham calibrator (using geomean of Actb and Gapdh housekeeping genes)
